# Left Head Rotation as an Alternative to Difficult Tracheal Intubation: Randomized Open Label Clinical Trial

**DOI:** 10.2196/42500

**Published:** 2023-08-04

**Authors:** Danya P Chan, George Carlos Rosendo M Jularbal III, Ismael Julius R Mapili

**Affiliations:** 1 Department of Anesthesiology Baguio General Hospital and Medical Center Baguio City Philippines

**Keywords:** tracheal, endotracheal, intubation, airway management, sniffing position, LeHeR

## Abstract

**Background:**

Tracheal intubation is a life-saving intervention, and optimizing the patient’s head and neck position for the best glottic view is a crucial step that accelerates the procedure. The left head rotation maneuver has been recently described as an innovative alternative to the traditional sniffing position used for tracheal intubation with marked improvement in glottic visualization.

**Objective:**

This study compared the glottic view and intubating conditions in the sniffing position versus left head rotation during direct laryngoscopy.

**Methods:**

This randomized, open-label clinical trial enrolled 52 adult patients admitted to Baguio General Hospital and Medical Center from September 2020 to January 2021 for an elective surgical procedure requiring tracheal intubation under general anesthesia. Intubation was done using a 45° left head rotation in the experimental group (n=26), while the control group (n=26) was intubated using the conventional sniffing position. Glottic visualization and intubation difficulty with the two procedures were assessed using the Cormack-Lehane grade and Intubation Difficulty Scale, respectively. Successful intubation is measured by observing a capnographic waveform in the end-tidal CO_2_ monitor after placement of the endotracheal tube.

**Results:**

There was no statistically significant difference in the Cormack-Lehane grade, with 85% (n=44) of patients classified under grades 1 (n=11 and n=15) and 2 (n=11 and n=7) in the left head rotation and sniffing position groups, respectively. In addition, there were no statistically significant differences in the Intubation Difficulty Scale scores of patients intubated with left head rotation or sniffing position; 30.7% (n=8) of patients in both groups were easily intubated, while 53.8% (n=14) in left head rotation and 57.6% (n=15) in sniffing position groups were intubated with slight difficulty. Similarly, there were no significant differences between the 2 techniques in any of the 7 parameters of the Intubation Difficulty Scale, although numerically fewer patients required the application of additional lifting force (n=7, 26.9% vs n=11, 42.3%) or laryngeal pressure (n=3, 11.5% vs n=7, 26.9%) when intubated with left head rotation. The intubation success rate with left head rotation was 92.3% versus 100% in the sniffing position, but this difference was not statistically significant.

**Conclusions:**

Left head rotation produces comparable laryngeal exposure and intubation ease to the conventional sniffing position. Therefore, left head rotation may be an alternative for patients who cannot be intubated in the sniffing position, especially in hospitals where advanced techniques such as video laryngoscopes and flexible bronchoscopes are unavailable, as is the case in this study. However, since our sample size was small, studies with a larger study population are warranted to establish the generalizability of our findings. In addition, we observed inadequate familiarity among anesthesiologists with the left head rotation technique, and the intubation success rate may improve as practitioners attain greater technical familiarization.

**Trial Registration:**

International Standard Randomised Controlled Trial Number (ISRCTN)ISRCTN23442026; https://www.isrctn.com/ISRCTN23442026

## Introduction

Tracheal intubation is an essential life-saving intervention. However, patient intubation in a difficult airway requires specialized technical skills, availability of appropriate equipment, and proper assessment of the clinical situation and priorities [1]. Consequently, experienced and inexperienced physicians or allied health professionals routinely encounter difficult intubation situations in the hospital and prehospital settings [2]. Moreover, predicting airway management-related difficulties remains a challenge and cause of frustration among anesthesiologists [3]. Although some studies have attempted to predict difficult intubation using a simple bedside physical examination [4], others have noted the limited and inconsistent capacity of bedside physical examination to identify patients with difficult airways [5]. Furthermore, assessing the risk of difficult airway intubation beforehand may be impossible during emergencies [6].

A study by Cheong et al [7] on airway practices suggested that standard airway examinations could predict only about half of the difficult intubations. Poor visualization of the larynx often leads to difficult intubation, which may result in complications such as aspiration, esophageal intubation, and prolonged hypoxia. Subsequently, these complications may increase patient morbidity and mortality [8]. Therefore, optimizing the patient's head and neck position for the best glottic view is crucial for successful tracheal intubation [9]. Achieving optimal head and neck position is also included in the Difficult Airway Society guidelines for managing adult patients with unanticipated difficult tracheal intubation [10].

Several head and body positions are used to facilitate tracheal intubation. The sniffing position, which is achieved by the flexion of the lower cervical spine, the extension of the upper cervical spine, and the extension of the atlanto-occipital joint [9], is the preferred position among anesthesiologists [11] and is the current gold standard in the intubation process [12]. Several studies have reported attaining an optimal head position for direct laryngoscopy and intubation with the normal airway in the sniffing position [9,12,13]. However, in some studies, the sniffing position did not improve glottic visualization, the success rate on first intubation, or intubation time [14,15]. These inconsistent findings with sniffing position pose a challenge for tracheal intubation in cases where alternate intubation techniques and devices, such as video laryngoscopes and flexible bronchoscopes, especially in low- and middle-income settings where advanced techniques may not be readily available in all hospitals. Therefore, anesthesiologists continuously explore other modalities to optimize the glottic view during direct laryngoscopy [16]. Consequently, various maneuvers have emerged as an alternative to the sniffing position, such as cricoid pressure application [17]; backward, upward, and rightward pressure [18]; head extension [19]; and external laryngeal manipulation [20].

Intubation in the lateral position has been especially well studied [21-24]. A systematic review of different intubation positions in trauma patients suggests reduced airway patency in the supine position compared to the lateral position [25]. In a supine position, the mechanisms of upper airway obstruction include reduction of pharyngeal dilator muscle activity and gravitational effects on anterior upper airway structures [26]. In contrast, lateral position widens the upper airway [27]; hence, upper airway obstruction can be significantly reduced to improve laryngeal visualization. Although some studies suggest that the lateral position may be more difficult than the supine position [28], a reduction in intubation time has been noted after the third attempt in the lateral position [29]. In a more recent study by Goh et al [30], patients were successfully intubated in the lateral position by anesthesiology trainees on the first attempt, with a mean duration of intubation of 57.3 (SD 36.4) seconds. The successful use of a video laryngoscope in the lateral position has also been previously reported [31]. Furthermore, some studies suggest that the head-elevated laryngeal position may be superior to the sniffing position [14,32], although the degree of head elevation necessary to facilitate the external auditory meatus and sternal notch alignment may vary among patients. Thus, Myatra [16] proposed abandoning the conventional “one size fits all” approach with headrests at a fixed height and considering an individualized intervention when positioning patients for laryngoscopy.

Adding to the range of available head and body positions to facilitate tracheal intubation, in 2019, Yezid et al [8] reported using the left head rotation maneuver to optimize head and neck position during tracheal intubation in nontrauma patients. Like the lateral position, left head rotation increases the upper airway's cross-sectional area due to the lateral displacement of the esophagus to the left of the cricoid cartilage. However, this lateral displacement of the esophagus has only been reported in awake nontrauma patients [33], while studies in sleeping subjects did not observe a decreased pharyngeal pressure with left head rotation [34].

Thus, whether head rotation improves airway patency and glottic visualization in anesthetized individuals remains uncertain. Therefore, in this randomized open-label clinical trial, we aimed to compare the glottic view and ease of intubation with left head rotation versus the conventional sniffing position during direct laryngoscopy of patients undergoing elective surgery and evaluate if the left head rotation maneuver is a viable alternative for difficult endotracheal intubation.

## Methods

### Research Design

This randomized open-label clinical trial enrolled patients admitted to Baguio General Hospital and Medical Center, Baguio City, Cordillera Administrative Region, the Philippines, from September 2020 to January 2021 for an elective surgical procedure requiring tracheal intubation under general anesthesia.

### Study Outcomes

The primary study outcome was intubation success rate with direct laryngoscopy using 45-degree left head rotation. Intubation was deemed successful if a capnographic waveform in the end-tidal CO_2_ monitor was observed after the endotracheal tube placement, and the intubation attempt was no longer than 10 minutes. Alternative techniques were used to facilitate that intubation in case intubation was unsuccessful with left head rotation or the sniffing position alone (Table 1). The order in which alternative techniques like cricoid pressure, stylet, and change in operator were used was left to the clinician's discretion. If the intubation was deemed unsuccessful after 2 attempts despite the use of alternative techniques, an alternative position was used (change to sniffing position if difficulty intubating with left head rotation, and vice versa).

**Table 1:** Description of the 7 parameters and scoring scheme of the Intubation Difficulty Scale.

Parameter	Score
N1: number of attempts >1	One point for every additional attempt if unsuccessful in the first attempt.
N2: number of operators >1	One point for each additional operator.
N3: number of alternative techniques^a^	One point for each alternative technique used.
N4: Cormack-Lehane grade	Zero points for successful intubation; otherwise, add Cormack-Lehane grade for the first attempt.
N5: lifting force required	Zero points for normal lifting force and 1 point for increased force.
N6: external laryngeal manipulation used	Zero points if not used and 1 point if used.
N7: vocal cord mobility	Zero points for abduction and 1 point for adduction.
Total IDS^b^ score	Sum of N1 to N7.

^a^Alternative techniques included the change of blade or tube, adding a stylet, changing to nasotracheal intubation, applying pressure on the cricoid cartilage, and using fiberoptic intubation or intubating laryngeal mask airway.

^b^IDS: Intubation Difficulty Scale.

### Sample Size

Due to limited studies with intubation using left head rotation, the sample size computation was based on the study by Khan et al [28], where the authors reported a 68% success rate in intubation with direct laryngoscopy using the left lateral position. Therefore, a sample size of 52, with 26 participants in each group, was computed using OPEN-EPI (version 3.1) with a 95% CI and 80% power, assuming a success rate of 68% with left head rotation and 100% with conventional intubation in the sniffing position.

### Inclusion or Exclusion Criteria

The criteria for inclusion in this study were patients aged 18-65 years old, BMI range of 18.5-35.0 kg/m^2^, American Society of Anesthesiology Physical Status I to III (see [35] for details of American Society of Anesthesiology Physical Status staging), and Mallampati grade III. Mallampati grade measures the visibility of pharyngeal structures (tonsillar pillars, soft palate, and base of uvula), which is noted by instructing the patient to open his or her mouth and protrude the tongue maximally in the sitting posture (see [36] for details of Mallampati grade classification).

Patients with sternomental distance <12 cm, thyromental distance <6 cm, small mouth opening <3 fingerbreadths, limited head rotation or neck extension, BMI >35 kg/m^2^, known gastroesophageal reflux, presence of anterior neck mass, or facial fractures obstructing the airway were excluded from this study.

### Randomization

**Figure 1:**
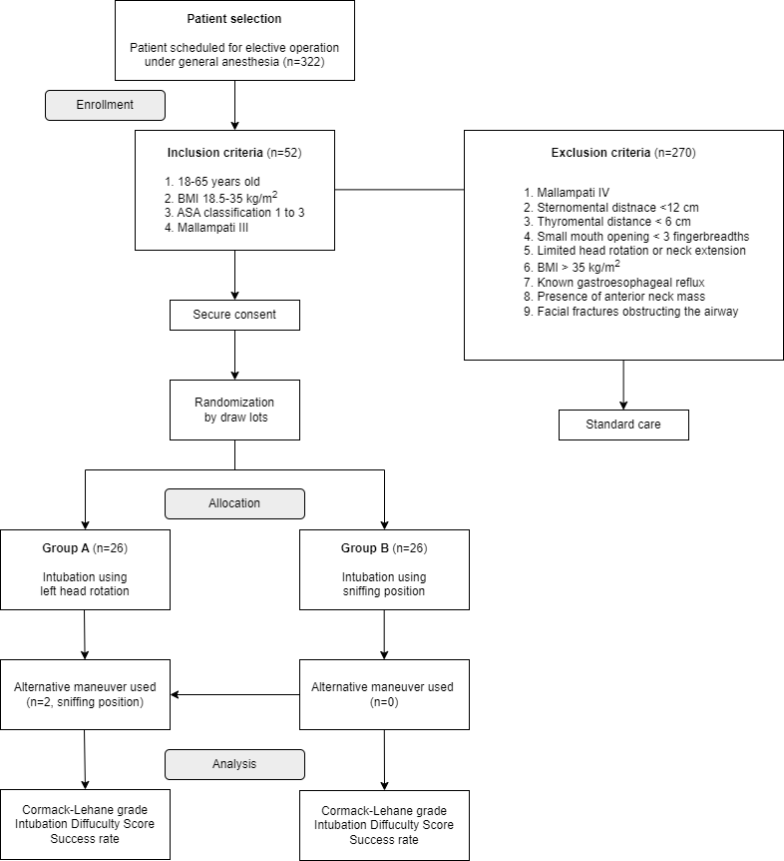
The flow of patient selection and randomization procedure. ASA: American Society of Anesthesiology.

Enrolled participants who met the inclusion criteria were randomized by draw lots into the experimental (intubated with left head rotation; n=26) and control groups (intubated in the sniffing position; n=26). Group assignments were written on a sheet of paper, which were either “group A” (left head rotation) or “group B” (sniffing position). The papers were shuffled for randomization and numbered for equal participant allocation to each group. The consultant or senior anesthesiology resident opened the papers drawn prior to the induction of anesthesia to determine group assignment. Thus, the consultant or senior anesthesiology resident served as the observer, and the researcher (DPC) was blinded during data collection to avoid bias. In addition, senior anesthesiologists who participated in the data collection were in year 2 or year 3 of their clinical residency. The flow of patient selection and randomization is described in Figure 1.

### Ethics Approval

The protocol and informed consent forms were reviewed and approved by the institutional ethics board of Baguio General Hospital and Medical Center (protocol BGHMC-ERC-2020-27). The researcher obtained written informed consent the day before the scheduled operation. The consent form was available in English, Filipino, and Ilocano, with identical content covering the nature of this study; study procedure; risks, benefits, and complications; data security and confidentiality; and voluntary participation and withdrawal. The contents of the consent forms were also verbally explained to the participants, and they were reminded that they were free to withdraw from this study at any point, and if they decided to withdraw prior to the surgical procedure, treatment quality would not vary, and standard care will be provided. The researcher also provided an audio-visual presentation of the intubation procedures in a manner or language that the patient, senior resident, and consultant understood. Several steps were taken to ensure the confidentiality and security of the data. Only DPC has access to the password-protected data, and upon completion of this study, all data were archived in the Hospital Information and Management System office for future reference.

### Intubation Procedure

The anesthesiology resident or consultant in charge performed a physical examination and a thorough airway evaluation during the preoperative evaluation to assess the ease of intubation. Laryngoscopy was done using an EMS Fiber Optic Laryngoscope Stubby Handle (EMS) throughout this study period with Macintosh Mega Mac Blade (EMS). Laryngoscope blades were disinfected with Caviwipes (Metrex Research LLC), washed with soap and water, and sterilized to prevent cross-contamination. Before intubation, the laryngoscope's functionality and battery status was checked by a senior resident.

Standard American Society of Anesthesiology monitors (electrocardiogram, noninvasive blood pressure, and pulse oximetry) were applied upon arrival at the operating room. Preprocedural medication included intravenous (IV) injections of midazolam (0.1 mg/kg) for anesthesia and fentanyl (2 mcg/kg) for analgesia. In addition, all patients were preoxygenated with 100% oxygen for 3 minutes through a circle system and a standard face mask with a carbon dioxide or flow sensor between the mask and the breathing circuit. Standard induction included injection of propofol at 2-2.5 mg/kg IV or until the loss of eyelash reflex was achieved and injection of rocuronium 0.6 mg/kg IV for muscle relaxation to facilitate intubation.

Macintosh number 3 or 4 laryngoscope blade was used depending on the anesthesiologist's decision. Intubation was performed with a tracheal tube size of 7.0 in women and 7.5 in men. Intubation was done using a 45-degree left head rotation (estimated with the aid of a protractor) in the experimental group, while the control group was intubated using a sniffing position by placing a cushion under the head such that the external auditory meatus and sternal notch are on the same horizontal plane. Glottic visualization and intubation difficulty with left head rotation and sniffing position were assessed using Cormack-Lehane grade [37] and Intubation Difficulty Scale [38], respectively, which were evaluated by the consultant or senior anesthesiology resident in charge (the researcher was not involved in the scoring).

Cormack-Lehane grade is a conventionally used scale that measures laryngoscopic or glottic view during laryngoscopy [39]. The 4 Cormack-Lehane grades are as follows: complete visualization of the vocal cords (grade 1), visualization of the inferior portion of the glottis (grade 2), visualization of only the epiglottis (grade 3), and nonvisualized epiglottis (grade 4). No external laryngeal pressure was applied for grading the laryngoscopic view [37].

The Intubation Difficulty Scale is an objective and comprehensive assessment of the intubation difficulty based on 7 parameters [38], as described in Table 1. A score of 0 on the Intubation Difficulty Scale represents ideal intubation with minimum difficulty, scores between 1 and 5 represent slight difficulty with intubation, and a score greater than 5 represents moderate to major difficulty with intubation (Table 1).

A carbon dioxide or flow sensor measured end-tidal carbon dioxide, the gold standard for confirming successful tracheal intubation. The airway was secured, and breaths were delivered through the endotracheal tube using an anesthesia ventilator by pressure-regulated volume control mode at 12 breaths per minute, inspiratory to expiratory ratio of 1:2, positive inspiratory pressure of 15 cm H_2_O, and positive end-expiratory pressure of 0 cm H_2_O. This study protocol ended at this point, and the intended surgical procedure proceeded as planned.

### Safety Considerations

The anesthesiologist in charge prioritized the patient's comfort and safety, and any changes in vital signs, such as hypotension and bradycardia, were actively monitored. Adequate hydration, oxygenation, and pain control were maintained throughout the procedure, and the risk of desaturation was minimized with 100% oxygen insufflation during laryngoscopy. Patient safety during apnea was ensured by continued physiological monitoring, including pulse oximetry in all cases. Although routine suction of secretions from the upper airways is not explicitly recommended, it was performed if symptoms suggestive of secretion accumulation were observed. The induction of the anesthetic, as well as the use of neuromuscular blocking agents, followed the latest anesthetic guidelines.

Injuries caused during difficult intubation were managed as follows: if a tooth was chipped or extracted, the patient's watchers were informed, and strict aspiration precautions were applied. Minor lacerations on the lips were allowed to heal via secondary intention, while large lacerations with persistent bleeding were sutured. Patients who failed to be intubated using left head rotation or standard sniffing position received an appropriate standard point of care based on Difficult Airway Society guidelines. An otolaryngologist or general surgeon was available if the procedure required invasive airway access, such as tracheostomy or cricothyrotomy. Untoward reactions were included in the report, and close follow-up was advised.

### Statistical Analysis

**Figure 2:**
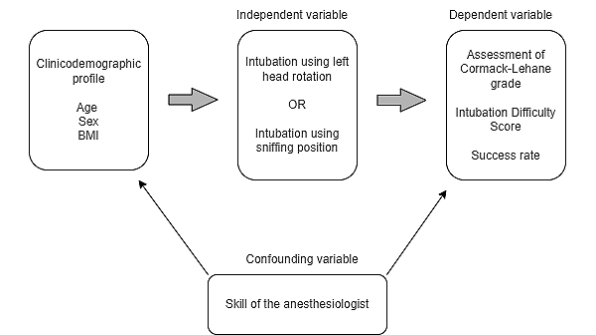
Conceptual framework: the relationship of variables in left head rotation.

Statistical analysis was conducted using SPSS (version 17.0; SPSS Inc). The conceptual framework for the analysis is described in Figure 2. Baseline characteristics, which included patient sex, age, and BMI, were presented as frequency and percentage, and the differences between experimental and control groups were compared using the chi-square test.

Noncontinuous variables, including the Cormack-Lehane grade (grade 1-4) and intubation difficulty (minimum, slight, and moderate to major) distribution of patients were presented as frequency and percentage, and the difference between the 2 study groups was assessed using the chi-square test. Additionally, the central tendency in the Cormack-Lehane grade of patients in the left head rotation and sniffing positions was presented as mean (SD) and median (IQR), and the difference between the 2 study groups was assessed using Student *t* test. Individual components (N1-N7) of the Intubation Difficulty Scale were presented as frequency and percentage (assessed using Fisher exact test) as well as mean (SD) and median (IQR) (assessed using Student *t* test). Finally, the intubation success rate was presented as frequency and percentage, and the difference between the 2 study groups was assessed using Fisher exact test. All tests were 2-sided, and *P* values of <.05 were considered statistically significant.

## Results

### Baseline Characteristics

In total, 52 adult patients were enrolled in this study; 57.7% (n=30) were male, and 55.8% (n=29) were 45 years or older (Table 2). The BMI of 50% (n=26) of the patients in both groups was in the normal range, while the rest were overweight or obese. No between-group differences were noted in the clinicodemographic characteristics of patients intubated with left head rotation or in the sniffing position (Table 2).

**Table 2:** Baseline characteristics of patients undergoing tracheal intubation with left head rotation or sniffing position (N=26).

Variables					Left head rotation			Sniffing position			*P* value^a^	
**Sex,** **n (%)**												.16
	Male			18 (69.2)			12 (46.1)					
	Female			8 (30.7)			14 (53.8)					
**Age (years), n (%)**												.71
	18-26			5 (19.2)			2 (7.6)					
	27-35			4 (15.3)			4 (15.3)					
	36-44			3 (11.5)			5 (19.2)					
	45-53			6 (23.0)			8 (30.7)					
	54-65			8 (30.7)			7 (26.9)					
**BMI**												.92
	**Normal (18.5-24.9 kg/m^2^)**											
		n (%)	13 (50)			13 (50)						
		Mean (SD)	22.8 (1.7)			22.26 (1.8)						
	**Overweight (25.0-29.9 kg/m^2^)**											
		n (%)	8 (30.7)			7 (26.9)						
		Mean (SD)	27.1 (1.4)			27.05 (1.4)						
	**Obese I (30.0-34.9 kg/m^2^)**											
		n (%)	5 (19.2)			6 (23.0)						
		Mean (SD)	32.4 (1.87)			32.14 (1.7)						

^a^Compared using the chi-square test. All values have been truncated to 1 decimal point.

### Glottic Visualization and Intubation Difficulty

Glottic visualization in nearly 85% (n=44) of the patients in the left head rotation and sniffing position was classified under grades 1 and 2 on the Cormack-Lehane grade scale. There was no significant association between Cormack-Lehane grade and the 2 intubation positions (*P*=.45; Table 3).

Further, 30.7% (n=16) of patients in both positions were intubated with minimum difficulty, 53.8% (n=14) in left head rotation and 57.6% (n=15) in sniffing position were intubated with slight difficulty, and moderate to major difficulty with intubation was noted in only a small number of patients in the 2 groups (n=4, 15.3% in left head rotation and n=3, 11.5% in sniffing position; Table 3). However, intubation difficulty was not significantly different between the 2 positions (*P*=.91; Table 3).

Although the proportion of patients with an Intubation Difficulty Scale score of 0-1 was numerically higher in the left head rotation group (n=18, 69.2% vs n=13, 50%), the difference was statistically insignificant (*P*=.26; Table 3). Similarly, the differences in median or median scores of the 7 variables of the Intubation Difficulty Scale were statistically insignificant between the 2 intubation positions (Table 3); the number of patients requiring more than one intubation attempt (N1; n=6, 23% vs n=5, 19.2% patients; *P*>.99), more than one operator (N2; n=3, 11.5% vs n=1, 3.8%; *P*=.61), or the use of alternate techniques for the successful passage of the endotracheal tube through the glottis (N3; n=7, 26.9% vs n=6, 23%; *P*>.99) was statistically not different between left head rotation and sniffing positions (Table 3). Similarly, the number of patients for whom the Cormack-Lehane grade for the unsuccessful first attempt was added to the total Intubation Difficulty Scale score (N4; n=14, 53.8% vs n=11, 42.3%; *P*=.50), required the application of additional lifting force (N5; n=7, 26.9% vs n=11, 42.3%; *P*=.38) or laryngeal pressure (N6; n=3, 11.5% vs n=7, 26.9%; *P*=.29) and displayed vocal cord mobility (N7; n=3, 11.5% vs n=2, 7.6%; *P*>.99) and was statistically not different between the 2 intubation positions (Table 3).

**Table 3:** Glottic visualization, intubation difficulty, and intubation success rate with left head rotation or sniffing position (N=26).

Outcomes				Left head rotation		Sniffing position		*P* value^a^
**Cormack-Lehane grade**								.45
	Grade 1, n (%)		11 (42.3)		15 (57.6)			
	Grade 2, n (%)		11 (42.3)		7 (26.9)			
	Grade 3, n (%)		3 (11.5)		4 (15.3)			
	Grade 4, n (%)		1 (3.8)		0 (0)			
	Mean (SD)		1.8 (2.3)		1.8 (1.7)		.95	
	Median (IQR)		1 (0-2.5)		1.5 (0-3)			
**Intubation difficulty, n (%)**								.91
	Minimum difficulty		8 (30.7)		8 (30.7)			
	Slight difficulty		14 (53.8)		15 (57.6)			
	Moderate to major difficulty		4 (15.3)		3 (11.5)			
Patients with IDS^b^ score of 0-1, n (%)				18 (69.2)		13 (50)		.26
**Individual IDS parameters**								
	**Patients with N1 score of >0**							
		n (%)	6 (23.0)		5 (19.2)		>.99	
		Mean (SD)	0.2 (0.4)		0.1 (0.4)		.73	
		Median (IQR)	0 (0-0.2)		0 (0-0)		—^c^	
	**Patients with N2 score of >0**							
		n (%)	3 (11.5)		1 (3.8)		.61	
		Mean (SD)	0.1 (0.3)		0.04 (0.2)		.30	
		Median (IQR)	0 (0-0)		0 (0-0)		—	
	**Patients with N3 score of >0**							
		n (%)	7 (26.9)		6 (23.0)		>.99	
		Mean (SD)	0.2 (0.4)		0.2 (0.4)		.74	
		Median (IQR)	0 (0-1)		0 (0-0.2)		—	
	**Patients with N4 score of >0**							
		n (%)	14 (53.8)		11 (42.3)		.58	
		Mean (SD)	0.7 (0.8)		0.5 (0.7)		.50	
		Median (IQR)	1 (0-1)		0 (0-1)		—	
	**Patients with N5 score of >0**							
		n (%)	7 (26.9)		11 (42.3)		.38	
		Mean (SD)	0.2 (0.4)		0.4 (0.5)		.26	
		Median (IQR)	0 (0-1)		0 (0-1)		—	
	**Patients with N6 score of >0**							
		n (%)	3 (11.5)		7 (26.9)		.29	
		Mean (SD)	0.1 (0.3)		0.27 (0.4)		.18	
		Median (IQR)	0 (0-0.2)		0 (0-0)		—	
	**Patients with N7 score of >0**							
		n (%)	3 (11.5)		2 (7.6)		>.99	
		Mean (SD)	0.1 (0.3)		0.08 (0.2)		.63	
		Median (IQR)	0 (0-0)		0 (0-0)		—	
Intubation success rate, n (%)				24 (92.3)		26 (100)		.49

^a^The Cormack-Lehane grade and intubation difficulty distribution were assessed using the chi-square test. The proportion of patients who scored >0 in individual IDS parameters was assessed using Fisher exact test. All mean (SD) were assessed using Student *t* test. All values have been truncated to 1 decimal point.

^b^IDS: Intubation Difficulty Scale.

^c^Not applicable.

### Intubation Success Rate

The intubation success rate was 100% in the sniffing position (Table 3). Two patients in the sniffing position classified under moderate to major difficulty on the Intubation Difficulty Scale were intubated successfully after the second attempt; hence, shifting position was deemed unnecessary.

On the other hand, 92.3% (n=24) of the patients were successfully intubated using left head rotation (*P*=.49 vs intubation rate in the sniffing position; Table 3). Three patients in the left head rotation were staged under moderate to major difficulty on the Intubation Difficulty Scale. Patient 1 had an Intubation Difficulty Scale score of 6 and had successful intubation after changing the operator on the second attempt. Patient 2 had a grade 3 glottic visualization and an Intubation Difficulty Scale score of 7 in the left head rotation position. Despite using a stylet, cricoid pressure, and additional lifting force, intubation was unsuccessful in this patient after 2 attempts. However, Cormack-Lehane grade improved to grade 2 and the Intubation Difficulty Scale score to 3 upon changing to the sniffing position. Patient 3 had grade 4 glottic visualization with an Intubation Difficulty Scale score of 8. The patient's airway could not be secured using left head rotation despite 2 intubation attempts, the use of a stylet, the application of cricoid pressure and additional lifting force, or the change of operator. After changing to the sniffing position, the Cormack-Lehane grade improved from grade 4 to grade 1, the Intubation Difficulty Scale score improved from 8 to 2, and intubation was successful on the first attempt.

## Discussion

### Principal Findings

Considering Mallampati III as a sensitive criterion for difficult intubation, the findings of this study suggest that endotracheal intubation with left head rotation can be achieved with comparable glottic visualization and difficulty to the conventional sniffing position in anesthetized patients undergoing elective surgery. However, it is worth noting that numerically fewer patients required the application of increased lifting force and laryngeal pressure when intubated with left head rotation, even though the differences in the 7 constituent parameters of the Intubation Difficulty Scale were statistically nonsignificant between patients intubated with left head rotation and sniffing position.

To our knowledge, this study is the first to comprehensively compare the effectiveness of left head rotation with the sniffing position as the primary technique used to facilitate tracheal intubation of anesthetized nontrauma patients undergoing elective surgery. Except for the case study by Yezid et al [8], which described the intubation of 4 patients using left head rotation, the effect of axial head rotation on airway patency has not been evaluated systematically. However, from our correspondence with the author (Dr Nur Hafiza Yezid, Emergency and Trauma Department, Hospital Jitra, Kedah, Malaysia; December 2019), we are aware of 2 ongoing studies using left head rotation: one being conducted at the Department of Anesthesiology, Ampang Hospital, Malaysia and the other at the Department of Emergency Medicine, University of Malaya, Malaysia. Unfortunately, the results of these investigations are yet to be published.

Nonetheless, prior studies have used variations of left head rotation in specific circumstances. For instance, Le Bervet et al [40] showed improved Cormack-Lehane grade score and intubation efficiency with a left-handed Macintosh blade when combined with a rotation of the cervical spine to the left in about 10% of patients under general endotracheal anesthesia. Similarly, Ueda et al [41] showed that adding left head rotation to the “ramped position” improved the laryngeal view compared to the ramped position alone. Head rotation is also recommended when performing cardiopulmonary resuscitation [42] and during drug-induced sleep endoscopy in patients with obstructive sleep apnea in the supine position [43].

Furthermore, difficult mask ventilation often coexists with difficult tracheal intubation. Two crossover clinical trials [26,44] have compared the efficiency of head rotation on face mask ventilation in patients requiring general anesthesia. Head rotation of 45° in anesthetized apneic adults significantly increased the efficiency of mask ventilation compared with the neutral head position [26]. On the other hand, a 30° clockwise lateral head rotation did not significantly affect mask ventilation volume [44]. It is noteworthy that both crossover clinical trials used right head rotation. However, because airway obstruction for most individuals is symmetric, rotation in the opposite direction is unlikely to alter the findings. In all these cases, intubation with head rotation was successful after more than one intubation attempt and in conjunction with other maneuvers (ramped position, sniffing position, supine position, hyperextension, and aid of a bougie).

The clinical experience of anesthesiologists performing endotracheal intubations may have played a significant role in our assessments of the difficulty of endotracheal intubation. Senior residents and consultants who participated in this study were oriented with the research process but had limited experience with left head rotation. Some awkwardness was noted during the first intubation attempt as residents performed intubation in the left head rotation position. The senior residents also noticed the need for greater familiarization with the left head rotation technique. Since the sniffing position is almost always the default approach, simulation training of left head rotation for practitioners is warranted to provide greater familiarization. Furthermore, regular use of the left head rotation technique in the future and documentation of challenges may help improve the intubation conditions with left head rotation. In this study, most residents noted some difficulty intubating with left head rotation during the first attempt, but intubation became easier during subsequent attempts with left head rotation. Left head rotation maneuver also complies with the Difficult Airway Algorithm recommended by the Difficult Airway Society. With more technical familiarity, it may be a practical noninvasive alternative approach to improve the glottic view among anesthetized patients requiring tracheal intubation. In addition, the potential outcome of this study can benefit patients by providing quicker airway access during intubations and fewer intubation attempts, thereby improving patient safety.

It is worth noting that while this study included patients who had Mallampati III classification during preoperative evaluations, only 8 out of the 52 patients enrolled in this study had a Cormack-Lehane grade of ≥3. Modified Mallampati classification is a widely used tool for predicting difficult airways, and a Mallampati score of III or IV is considered a good predictor of difficult intubation [45,46]. For instance, previous studies by Adnet et al [47] and Oria et al [48,49] showed greater difficulty in intubating patients with Mallampati III and IV, decreased thyromental distance, reduced mouth opening or other anatomical abnormalities than patients without any predictive factors of intubation difficulty. Even though moderate to major difficulty is infrequent in earlier reports and observed in only about 8% of the patients, the rate of intubation with any problem is surprisingly low [47]. However, the Mallampati classification has exceedingly high specificity when used alone, but the sensitivity is typically low, with an increased number of false-positive results [46,50]. While multiple indicators have been identified for predicting difficult airway [4,50] and a single specific technique would be ideal for a quick and easy assessment, the observation in this study supports the findings of previous studies that Mallampati classification, when used solely, may not have adequate sensitivity in predicting difficult laryngoscopy, intubation, or bag-valve-mask ventilation [46,51].

### Limitations

There are several limitations of this study. First, this study was conducted over a short timeframe and may have lacked adequate population representation. Second, although we included adequate participants assuming a success rate of 68% with left head rotation, the sample was not large enough to achieve statistical significance when the changes were minor. Therefore, more extensive trials with a larger and more diverse study population are needed to establish the effectiveness of left head rotation or lack thereof. These limitations prevented us from making firm conclusions on some study outcomes. For instance, all patients were successfully intubated in the sniffing position, while 2 patients in the left head rotation required changing to the sniffing position for successful intubation. Therefore, more than one attempt at intubation, the need for more than one operator, and using an alternative technique such as a stylet were more common in the left head rotation group. Although these results indicate that the sniffing position may provide better laryngeal exposure and intubation ease than left head rotation, the small number of patients with the outcome prevents us from drawing a firm conclusion on the superiority of the sniffing position.

Third, given the scarcity of evidence to support the use of left head rotation as a maneuver to optimize tracheal intubation, this study was limited to a patient population where a minimal delay to the intubation period would not present a significant risk to the subject, further limiting the generalizability of our findings. Fourth, this study was conducted during the COVID-19 pandemic, and level 4 personal protective equipment may have influenced the intubation techniques. Studies even before the pandemic have identified the practical problems of excessive heating and fogging while wearing a transparent face shield device during tracheal intubation of patients, although personal protective equipment had no significant effect on the intubation time [52]. Fifth, since this study is a randomized, open-label clinical trial, the observer could not be blinded due to apparent differences in head positions. Lastly, proper airway evaluation and visualization can be affected by the skill of the anesthesiologist, which was not factored in our analysis as all of them had limited experience with left head rotation. In contrast, they all had extensive experience with the sniffing position, which could have confounded our findings.

### Conclusions

This study showed that left head rotation produces comparable laryngeal exposure and intubation ease to the conventional sniffing position. Therefore, left head rotation may be an alternative for patients who cannot be intubated in the sniffing position, especially in hospitals where advanced techniques such as video laryngoscopes and flexible bronchoscopes are unavailable, as is the case in this study. Since the sniffing position is used as the default, it remains plausible that better clinical outcomes may be achieved with the left head rotation technique as practitioners attain better technical familiarization. Studies with a larger study population are warranted to establish the generalizability of our findings.

## Appendix

Multimedia Appendix 1 CONSORT-eHEALTH checklist (V 1.6.1).

## Abbreviations

IV: intravenous

## Data Availability

The data sets generated and analyzed during this study are available from the corresponding author upon reasonable request.
